# Effects and Mechanisms of Long-Term *Lycium barbarum* Water Consumption on Skeletal Muscle Function in Aged Mice

**DOI:** 10.3390/foods14173049

**Published:** 2025-08-29

**Authors:** Yundi Tang, Qingwei Zheng, Jinyi Wang, Mingcong Fan, Haifeng Qian, Li Wang, Yan Li

**Affiliations:** State Key Laboratory of Food Science and Resources, School of Food Science and Technology, Jiangnan University, Wuxi 214122, China; 1013230104@stu.jiangnan.edu.cn (Y.T.); 6220112120@stu.jiangnan.edu.cn (Q.Z.); 1013230203@stu.jiangnan.edu.cn (J.W.); fanmc@jiangnan.edu.cn (M.F.); qianhaifeng@jiangnan.edu.cn (H.Q.); wangli@jiangnan.edu.cn (L.W.)

**Keywords:** *Lycium barbarum* water, skeletal muscle aging, oxidative stress, inflammation, aging-related factors, metabolomics analysis

## Abstract

With the global aging population, skeletal muscle aging has threatened to elderly health, making dietary interventions for age-related muscle decline a research priority. *Lycium barbarum*, a traditional food and medicinal herb, was used in the study to prepare *Lycium barbarum* water (LBW). This experiment was conducted in animals and included four groups: young control (C-Young), aged control (C-Aged), young LBW-drinking (G-Young), and aged LBW-drinking (G-Aged). Assessments covered skeletal muscle mass, cross-sectional area, and exercise ability to compare health status. The study measured mRNA expression of Atrogin-1 and MuRF-1 from the Forkhead Box O (FOXO) pathway, advanced glycation end products (AGEs) and senescence-associated β-galactosidase (SA-β-gal), oxidative stress levels via superoxide dismutase (SOD), malondialdehyde (MDA) and glutathione (GSH), inflammatory levels through interleukin-10 (IL-10) and tumor necrosis factor-alpha (TNF-α), and applied untargeted metabolomics to profile metabolic alterations. Optimal LBW was achieved at 80 °C with a 1:10 (*w*/*v*) solid-liquid ratio. In aged mice, long-term LBW administration improved exercise capacity, reduced muscle atrophy, and increased muscle mass, alongside decreased aging-related markers, alleviated oxidative stress, and modulated inflammatory levels. Additionally, metabolomics confirmed age-related oxidative stress and inflammation. Long-term LBW consumption alleviates age-related skeletal muscle dysfunction via multi-target regulation, holding promise as a natural nutritional intervention for mitigating skeletal muscle aging.

## 1. Introduction

Aging is an inevitable biological process characterized by progressive functional decline across multiple organ systems, laying the foundation for chronic diseases [1]. The global population is aging rapidly, with adults aged over 65 years old projected to account for 16% of the world’s population by 2050 [2]. This demographic shift has heightened interest in nutritional interventions to delay age-related tissue degeneration, particularly in skeletal muscle—the body’s largest metabolic organ [3].

Skeletal muscle aging is marked by reduced muscle fiber cross-sectional area, impaired motor function, and increased fall risk [4,5]. Beyond mobility issues, sarcopenia triggers metabolic disorders such as type 2 diabetes and dyslipidemia [6], forming a vicious cycle driven by FOXO-mediated myofiber breakdown and NF-κB-induced chronic inflammation [7,8]. Epidemiologically, sarcopenia affects 10–27% of Europeans aged ≥60 and up to 41.0% of Asians aged ≥65, highlighting the urgency for effective nutritional strategies [9,10].

Natural products have emerged as promising anti-aging agents due to their multi-target effects and safety. Goji (*Lycium barbarum*), extensively cultivated in China, is primarily found in the northwestern and northern regions [11]. This plant, known for its dual medicinal and edible uses, boasts a medicinal history of over two millennia and holds a prestigious place in Chinese medicine [12]. Modern research has identified that *Lycium barbarum* is rich in *Lycium barbarum* polysaccharides (LBP, comprising 5–8% of its content), carotenoids, betaine, and polyphenolic compounds, which offer various benefits such as immune enhancement, anti-cancer activity, hypoglycemic, hypotensive, hypolipidemic effects, anti-aging, and cosmetic advantages [13,14,15]. Additionally, *Lycium barbarum* fruits significantly boost muscle and hepatic glycogen storage, enhance human vitality, and exhibit anti-aging properties [16,17,18]. While *Lycium barbarum* is an economically significant cash crop, current research on it has largely focused on extracts of specific bioactive components such as LBP rather than natural aqueous preparations [19,20]. However, focusing on single-component extracts may miss synergies among multiple bioactive substances, as natural products act via combined effects. This has resulted in a relative paucity of studies regarding the comprehensive anti-aging effects of LBW.

To address this gap, the study aims to optimize the preparation protocol for *Lycium barbarum* water (LBW), using mice as models, and conduct long-term interventions by replacing daily drinking water with LBW in both young and aged mice groups. LBW aligns with traditional use—goji is commonly consumed via boiling/soaking, retaining a comprehensive profile of native components without losses or alterations from complex extraction (e.g., solvent use) [21]. It also avoids residual reagent risks, suiting long-term dietary interventions that simulate real consumption. Furthermore, the present study focuses on the combined effects of multiple components in LBW, and this perspective further enhances the novelty of the research. The study monitored changes in various indexes, assessed motor capacity and skeletal muscle atrophy-related parameters, and explored the effects of long-term LBW consumption on skeletal muscle function and its underlying mechanisms, focusing on oxidative stress, inflammation, and metabolic pathways that drive skeletal muscle aging. This study not only enhances understanding of LBW’s anti-aging potential but also provides a theoretical basis for developing natural nutritional interventions to mitigate skeletal muscle aging and improve elderly health, thus holding significant theoretical and practical importance.

## 2. Materials and Methods

### 2.1. Materials

Young (8-week-old) and aged (18-month-old) male C57BL/6J mice were purchased from Vital River Laboratories (Beijing, China). Dried *Lycium barbarum* fruits (Ningxia origin) were obtained from official flagship store of Ningxia Red. Chromatographic grade methanol (batch No.: 10014108, purity ≥ 99.7%), chromatographic grade acetonitrile (batch No.: 400641646, purity ≥ 99.9%), analytical grade formic acid (batch No.: XW06418606, purity ≥ 99%), analytical grade phosphoric acid (batch No.: 100154008), analytical grade trichloromethane (batch No.: 10006818, purity ≥ 99.0%), analytical grade chloroform (batch No.: HW049401, purity ≥ 99.9%), and analytical grade absolute ethanol were all purchased from Sinopharm Chemical Reagent Co., Ltd. (Shanghai, China). ELISA kits for the detection of advanced glycation end products (AGEs, batch No.: EHJ-30217m), senescence-associated β-galactosidase (SA-β-gal, batch No.: EHJ-30337m), superoxide dismutase (SOD, batch No.: EHJ-30396m), malondialdehyde (MDA, batch No.: EHJ-30395m), glutathione (GSH, batch No.: EHJ-98364m), interleukin-10 (IL-10, batch No.: EHJ-47391m), and tumor necrosis factor-α (TNF-α, batch No.: EHJ-45111m) were supplied by Huijia Biotechnology Co., Ltd. (Shanghai, China). Hematoxylin was obtained from YD Diagnostics (Seoul, Republic of Korea), and the frozen section staining kit (batch No.: 99-900-01) was purchased from Thermo Fisher Scientific (Waltham, MA, USA).

The main instruments and equipment used in this study included: adjustable-volume pipettes (Model: Research plus) from Eppendorf AG (Hamburg, Germany); a high-speed refrigerated centrifuge (Model: CR-GIII) from Hitachi, Ltd. (Tokyo, Japan); a decolorizing shaker (Model: TS-100), a vacuum pump (Model: GM-0.33A), and a vortex oscillator (Model: Vortex-Genie 2) from Kylin-Bell Lab Instruments Co., Ltd. (Haimen, China); a double-beam UV-visible spectrophotometer (Model: A560) from AOE Instruments Co., Ltd. (Shanghai, China); a BIO-GS3 grip strength meter from BIOSEB (Vitrolles, France); a ZB-200 rotarod fatigue tester from Chengdu Techman Software Co., Ltd. (Chengdu, China); a platform treadmill (Model: Treadmill for Mice, Cat. No. 39250) from Noldus Information Technology Co., Ltd. (Beijing, China); and a fully automatic microplate reader (Model: SH-1000LAB) from Corona Electric Co., Ltd. (Ibaraki, Japan).

### 2.2. Preparation of LBW

The preparation of LBW was modified from the method described by Zhou et al. [22], using a single-factor optimization design to determine optimal conditions. The primary optimization objectives were to maximize antioxidant activity (see Section 2.3) and the content of major bioactive components (see Section 2.4). Two independent variables were evaluated: solid-liquid ratio (*Lycium barbarum*:distilled water, *w*/*v*) at four levels (1:2, 1:5, 1:10, 1:20) and extraction temperature at four levels (40 °C, 60 °C, 80 °C, 100 °C). All other parameters were kept constant: extraction duration (1 h in a water bath), ultrasonic-assisted extraction (30 min, fixed power 300 W, frequency 40 kHz), centrifugation (5000 rpm for 10 min), and re-extraction of residues under identical conditions to ensure maximum component yield. Each combination of ratio and temperature was tested in triplicate, using 100 g batches of cleaned *Lycium barbarum* (uniform in size and maturity to minimize variability). Briefly, mixtures were heated, subjected to ultrasonic extraction, centrifuged, and filtered. Residues were re-extracted, and combined supernatants were concentrated to half the original volume via rotary evaporation. The resulting LBW was sterilized (autoclaved at 121 °C for 20 min) and stored at 4 °C until subsequent experiments.

### 2.3. Antioxidant Properties

The antioxidant activity of LBW was estimated by superoxide radical, 2,2-Diphenyl-1-picrylhydrazyl (DPPH) and hydroxyl radical scavenging activity, as described in the previous study [23,24]. In brief, 100 μL of LBW at various concentrations was mixed with 100 μL of 0.2 mM DPPH solution in methanol. The mixture was incubated in the dark for 30 min at room temperature, and the absorbance was measured at 517 nm. The scavenging activity was calculated using the Equation (1).

(1) $$\mathrm{DPPH}\;\mathrm{radical}\;\mathrm{scavenging}\;\mathrm{activity}(\%)=(1-\frac{A_{experiment}}{A_{control}})\times 100$$

ABTS radical cations were generated by reacting 7 mM ABTS solution with 2.45 mM potassium persulfate and incubating in the dark for 16 h. The ABTS solution was diluted with ethanol to an absorbance of 0.70 ± 0.02 at 734 nm. Then, 10 μL of LBW was mixed with 190 μL of the diluted ABTS solution, and the absorbance was recorded after 6 min. The scavenging activity was calculated using the Equation (2).

(2) $$\mathrm{ABTS}\;\mathrm{radical}\;\mathrm{cation}\;\mathrm{scavenging}\;\mathrm{activity}(\%)=(1-\frac{A_{experiment}}{A_{control}})\times 100$$

The hydroxyl radical scavenging reaction mixture containing 50 μL of 9 mM FeSO_4_, 50 μL of 9 mM salicylic acid-ethanol solution, 50 μL of LBW, and 50 μL of 8.8 mM H_2_O_2_ was incubated at 37 °C for 30 min. The absorbance was measured at 510 nm. The scavenging activity was calculated as Equation (3).

(3) $$\mathrm{Hydroxyl}\;\mathrm{radical}\;\mathrm{clearance}(\%)=\frac{A_{water}-(A_{sample}-A_{control})}{A_{water}}\times 100$$

### 2.4. Component Analysis of LBW

Following the selection of the optimal LBW ratio with the highest radical scavenging activity, the basic chemical. composition of the extract was further analyzed. Analytical methods polysaccharides in LBW were quantified via phenol-sulfuric acid colorimetry (λ = 490 nm), following microwave-assisted extraction and glucose calibration. Anthocyanins were extracted with acidified ethanol (0.1% HCl) and analyzed by HPLC-C18 (520 nm). Total phenolics used Folin-Ciocalteu assay (765 nm), while total flavonoids employed NaNO_2_-Al(NO_3_)_3_ colorimetry (495 nm). Carotenoids were separated on a C30 column (450 nm) after hexane-acetone extraction. Betaine underwent methanol extraction, Alumina B SPE purification, and HILIC-NH2 HPLC (acetonitrile-water 85:15, 195 nm) [25].

### 2.5. Animal Experiment

The animal experiment protocol involved in this study was reviewed and approved by the Laboratory Animal Ethics Committee of Jiangnan University (Wuxi, China), with the approval number JN. No 20240430c0600930[205]. Experiments were conducted in a barrier environment with strict control of temperature at 25 ± 2 °C, relative humidity at 55 ± 5%, and a 12-h light/dark cycle. According to Mitchell, mice aged 18 months were defined as aged mice [26]. In this experiment, young mice (8 weeks old) and naturally aged mice (≥18 months old) were selected, and divided into four groups: Young Control Group (C-Young), Aged Control Group (C-Aged), Young LBW Group (G-Young), and Aged LBW Group (G-Aged). The absence of a positive control was deliberate: our focus is LBW’s intrinsic effects as a natural dietary intervention, not efficacy comparisons with known agents. The four-group design suffices—C-Young vs. C-Aged reveals age-related muscle changes; intragroup comparisons quantify LBW’s effects on each age group. This aligns with 3R principles (reducing animal use) and avoids confounding from mechanistic differences between LBW (food-derived) and positive controls like pharmaceuticals, ensuring scientific rigor and ethical compliance.

Specifically, mice were first stratified by age (young/aged); within each stratum, individuals were allocated to the corresponding control (Group C) or LBW (Group G) subgroup using a random number generator to balance baseline characteristics such as body weight. Outcome assessment was performed by researchers blinded to group assignments to minimize bias. The sample size in this study (*n* = 8 per group) was determined with reference to previous studies on mice models of skeletal muscle aging. A too small sample size may lead to the specificity of the sample, while an excessively large sample size is not in line with animal ethics. Through comprehensive analysis, it was confirmed that a sample size of 8 mice per group is sufficient to detect significant differences [27,28]. Mice were housed in cages at a density of 4 mice per cage (Table 1). All mice had free access to food and water throughout the trial. In the G-Young and G-Aged groups, drinking water was replaced with LBW, which was renewed every 2 days to ensure quality, and the total intervention period lasted 12 weeks. A 12-week intervention was selected based on two key considerations: it aligns with the regenerative cycles of skeletal muscle stem cells (about 2 weeks per cycle), ensuring sufficient duration to observe cumulative effects on muscle homeostasis [29]; and it approximates long-term human consumption patterns when scaled via the mouse-to-human lifespan ratio, where 12 weeks in mice corresponds to about 10 years of regular intake in humans, a timeframe consistent with dietary intervention studies on age-related muscle decline [30].

During the experiment, body weight of mice was recorded weekly, and food/water intake per cage was measured and normalized to per-mouse consumption. At the end of the experiment, mice were first anesthetized via inhalation of 1–1.5% isoflurane followed by blood collection from the orbital venous plexus, and after blood sampling, mice were euthanized by cervical dislocation. Collected blood was centrifuged at 3500 r/min for 15 min to isolate serum, which was stored at −80 °C for subsequent analysis. After taking gastrocnemius muscle (GAS), Tensor Fasciae Latae (TA) and Soleus (SOL), all other samples were washed and then transferred into 1.5 mL centrifuge tubes, rapidly frozen in liquid nitrogen, and stored at −80 °C for subsequent use.

### 2.6. Section of Skeletal Muscles and Hematoxylin and Eosin-Straining

GAS was embedded in FSC 22 clear frozen section embedding medium, rapidly frozen in liquid nitrogen-cooled isopentane, and stored at −80 °C for subsequent experiments [31]. Each muscle was sectioned into 8-μm-thick continuous horizontal slices using a cryostat maintained at −20 °C, with a portion of the mid-belly circumference selected for processing. The sections were stained with hematoxylin and eosin bought from Sigma-Aldrich, Inc. (St. Louis, MO, USA), then observed and imaged under a microscope. The average muscle fiber size was quantified by measuring the cross-sectional area of at least 100 randomly selected muscle fibers from no fewer than three sections per muscle. Fiber boundaries were manually outlined with a calibrated pen, and the area was automatically calculated using ImageJ software.

### 2.7. Mice Grip Strength Test

The forelimb grip strength test is recognized as the preferred non-invasive method for evaluating sarcopenia, as it measures the maximum contractile force during the animal’s spontaneous activity [32]. Referencing the grip strength test protocol by Lauretani, mice were positioned on a small-animal grip strength meter, allowing their forelimbs to firmly grasp the sensor [33]. The tail was then pulled horizontally backward at a constant speed to measure the pulling force. Each mouse underwent three trials, and the maximum value was recorded.

### 2.8. Mice Rotarod Fatigue Test

The rotarod test serves as a critical method to assess balance and coordination in sarcopenic mice. In this experiment, mice were placed on a rotating rod, and the duration of walking or maintaining balance on the rod, as well as the number of falls, were recorded to evaluate motor capacity and coordination [34]. Prior to formal testing, mice underwent a 3-day acclimation period during which they were placed on the rotarod with the speed increasing uniformly from 5 rpm to 10 rpm over 5 min each day. During the formal measurement, mice were placed on the rotarod set at 30 rpm, and the time until they fell off was recorded for each of three trials per mouse.

### 2.9. Mice Treadmill Exercise Test

Mice were first acclimated to the treadmill for 3 days at a constant speed of 5 m/min for 15 min daily; during the formal test, the treadmill was set to an initial speed of 5 m/min with a 1 m/min acceleration until the mice reached exhaustion, at which point the total running distance and time were recorded to assess exercise capacity.

### 2.10. Measurement of Expression Levels of Aging-Related Factors

The contents of SA-β-gal and AGEs were determined using commercial kits from Huijia Biotechnology, China, following the manufacturer’s instructions. Briefly, for SA-β-gal detection, mouse skeletal muscle tissue sections or cultured cells were fixed with 4% paraformaldehyde at room temperature for 15 min, rinsed with PBS, and then incubated with a staining solution containing X-gal substrate (pH 6.0) at 37 °C in the dark for 12–16 h. The proportion of blue-positive cells (characteristic staining of senescent cells) was observed under an optical microscope, or enzyme activity was quantified using a fluorescence probe method. For AGEs measurement, serum or tissue homogenates (prepared by homogenizing tissues in 1:9 ice-cold saline, centrifuging at 12,000× *g* for 20 min at 4 °C) were diluted appropriately and analyzed using a double-antibody sandwich ELISA. Samples and standards were added to 96-well plates coated with specific antibodies, followed by incubation with enzyme-labeled secondary antibodies, washing steps, and color development with TMB substrate. Absorbance was measured at 450 nm, and AGEs concentrations were calculated from standard curves. Tissue results were normalized to total protein content, while serum results were expressed as ng/mL. All procedures strictly adhered to the kit protocols to ensure accurate assessment of senescence markers.

### 2.11. Measurement of Oxidative Stress and Inflammatory Levels

Oxidative stress markers (SOD, MDA, GSH) and inflammatory cytokines (IL-10, TNF-α) in serum and skeletal muscle were measured using commercial ELISA kits bought from Huijia Biotechnology Co., Ltd. (Shanghai, China) following the protocol described by Wang [35]. Briefly, serum samples were prepared by centrifuging blood at 3500× *g* for 15 min at 4 °C, while muscle tissues were homogenized in ice-cold saline (1:9 *w*/*v*), centrifuged at 12,000× *g* for 20 min, and supernatants collected. Samples were diluted (1:5–1:10) to ensure readings within the standard curve range. Assays were performed according to the kit instructions, including incubation with specific antibodies, washing steps, and colorimetric detection at 450 nm. Results were normalized to total protein content (BCA assay) for tissues and expressed as ng/mL for serum. All samples were analyzed in duplicate, and the intra-assay coefficient of variation was <10% for quality control.

### 2.12. Quantitative Real-Time PCR Analysis (qRT-PCR)

Specific experimental procedures were referenced from Pan [36]. Briefly, RNA was extracted from skeletal muscle tissues using TRIzol reagent (Invitrogen, Carlsbad, CA, USA), followed by cDNA synthesis with the Prime Script RT system (Takara Bio Inc., Kyoto, Japan). After cDNA synthesis, the expression levels of the mRNA were also measured via the ABI 7900 system, using 18S rRNA as the internal reference. The qRT-PCR primer sequences are listed in Table 2.

### 2.13. Western Blot

Muscle proteins (30 μg) were separated by 10% SDS-PAGE, followed by transfer onto PVDF membranes. The membranes were probed with anti-Atrogin-1 (1:1000), anti-MuRF-1 (1:1000), and anti-β-actin (1:5000) antibodies, respectively. Protein bands were visualized using an ECL detection system and quantified with ImageJ 1.54 (National Institutes of Health, Bethesda, MD, USA).

### 2.14. Unsupervised Metabolomics Analysis

Metabolomics analysis is mainly done by institutions, and the specific steps are as follows. For sample preparation, quality control (QC) samples were prepared by pooling equal volumes of all experimental serum samples and processed using the same protocols as the test samples and 120 μL aliquots of frozen serum were mixed with 480 μL of an extraction solution composed of methanol and acetonitrile (2:1 *v*/*v*) [37]. The extraction solution included two standards: 2-chloro-L-phenylalanine and decanoic acid. Samples were vortexed for 120 s and then incubated at 4 °C for 30 min. After centrifugation at 14,000 r/min for 10 min, the supernatant was divided into two 250 μL aliquots: one for immediate analysis and the other stored for subsequent use. The aliquot designated for analysis was evaporated to dryness using a Labconco Centrivap Console, reconstituted in 125 μL of 50% methanol, and recentrifuged at 14,000 r/min for 10 min. The resulting supernatant was transferred to a 200 μL MicroSert Insert for analytical processing. Batch correction was performed using a QC sample-driven median centering algorithm, validated by PCA to eliminate technical variation while preserving biological differences. The final data was exported and analyzed and plotted with Majorbio Cloud Platform (Shanghai Majorbio Bio-pharm Technology Co., Ltd., Shanghai, China).

### 2.15. Statistical Analysis

Statistical and significance analyses were performed using GraphPad Prism 8.0 software (GraphPad Software, LLC, San Diego, CA, USA). All experiments were repeated at least three times, and results are presented as the mean ± standard error of the mean (SEM). Differences between two groups were determined using Student’s *t*-test, while differences among means of three or more groups were analyzed by one-way analysis of variance (ANOVA). Statistical significance was set at *p* < 0.05, and significant differences were indicated by different letters (*p* < 0.05).

## 3. Result

### 3.1. Optimization of LBW Preparation

Systematic investigation of the effects of different solid-to-liquid ratios and extraction temperatures on the antioxidant activity of LBW revealed that an extraction condition of 1:10 (*w*/*v*) solid-to-liquid ratio at 80 °C yielded the highest scavenging capacities against DPPH, ABTS, and hydroxyl radicals (Table 3 and Table 4). Statistical analysis confirmed that this extraction condition not only exhibited significantly higher radical scavenging rates compared to other experimental groups but also demonstrated minimal data dispersion, indicating excellent stability (*p* < 0.05). These results suggest that the antioxidant activity of LBW is maximally enhanced under these parameters, enabling efficient scavenging of biological radicals and showcasing superior antioxidant performance.

After the extraction process of LBW was determined, the specific components of LBW under the process were further measured (Table 5). It revealed high contents of key bioactive components. The most active ingredient polysaccharide also matches that mentioned by Masci [38]. These components not only serve as the material basis for LBW’s antioxidant properties but also provide a robust guarantee for the biological activity of LBW used in subsequent animal experiments.

### 3.2. Effects of Long-Term LBW Consumption on Mice Motor Function and Body Weight

**Figure 1:**
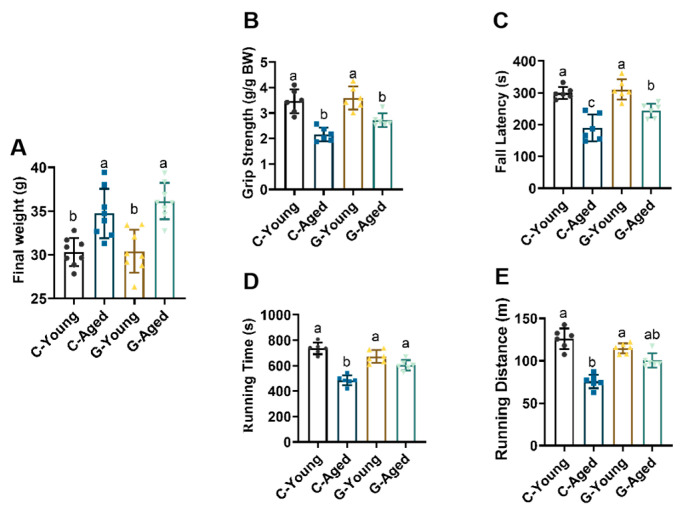
Effect of long-term LBW consumption on weight and exercise ability in mice: (**A**) Final weight (*n* = 8); (**B**) Grip strength, (**C**) Rotary rod drop time, (**D**) Running time, (**E**) Running distance (*n* = 6 for **B**–**E**). Significant differences were indicated by different letters (*p* < 0.05).

From Figure 1A, results showed that aged mice generally had higher body weights than young mice; however, there were no significant difference between the C-Aged and G-Aged groups (*p* = 0.6087), nor between the C-Young and G-Young groups (*p* = 0.9998). Thus, it is concluded that LBW had no direct promoting or inhibitory effect on mouse body weight regulation.

Motor function was assessed via grip strength, fall latency, and treadmill performance tests. As shown in Figure 1, LBW consumption had no significant effect on motor indices in young mice (for grip strength, *p* = 0.9269; for fall latency, *p* = 0.9291; for running time, *p* = 0.0889; for running distance, *p* = 0.1620). In contrast, the G-Aged exhibited significantly higher mean grip strength, longer rotarod fall latency, and greater treadmill running time and distance compared to the C-Aged (*p* < 0.05). These findings indicate that long-term LBW intake significantly improves muscle function in aged mice, enhancing both muscle strength and exercise endurance.

### 3.3. Effects of Long-Term LBW Consumption on Skeletal Muscle Mass in Mice

**Figure 2:**
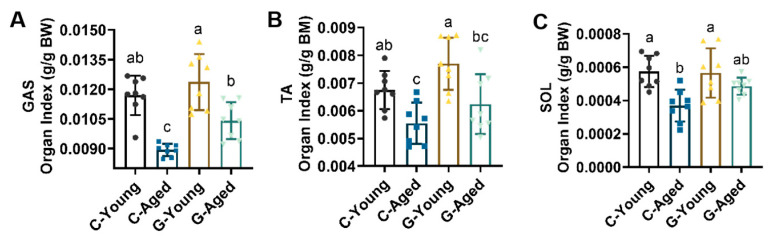
Effects of long-term LBW consumption on organ indexes of (**A**) GAS; (**B**) TA; (**C**) SOL (*n* = 8). Significant differences were indicated by different letters (*p* < 0.05).

Skeletal organ indexes were measured as shown in Figure 2. The C-Aged group showed significantly reduced indices for gastrocnemius (GAS), tibialis anterior (TA), and soleus (SOL) muscles compared to C-Young (for GAS, *p* < 0.0001; for TA, *p* = 0.0483; for SOL, *p* = 0.0037). Conversely, the G-Aged group displayed a significant increase in TA and SOL organ indexes approaching those of young healthy mice when compared to C-Aged. These results indicate that long-term LBW consumption significantly enhances skeletal organ indexes in aged mice, contributing to the maintenance of physical health, whereas its effects on muscle function in young mice are negligible.

**Figure 3:**
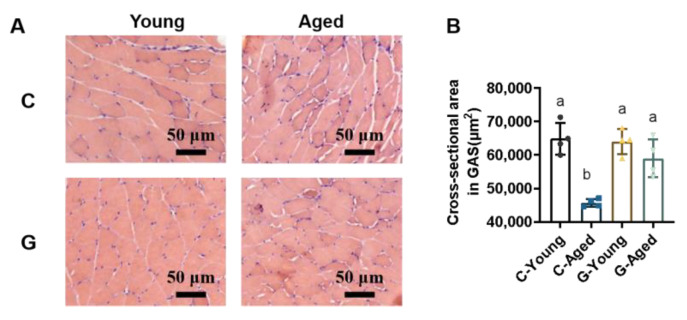
Effects of long-term LBW consumption on GAS muscle fiber cross-sections in mice (*n* = 4): (**A**) Representative GAS muscle fiber cross-sections; (**B**) GAS muscle cross-sectional area. Significant differences were indicated by different letters (*p* < 0.05).

Skeletal muscle cross-sectional morphology was observed via H&E staining, with quantitative analysis of GAS muscle fiber CSA (Figure 3). In young mice, muscle fibers were tightly arranged and morphologically intact. In contrast, the C-Aged group exhibited significantly reduced GAS fiber CSA (*p* = 0.0001), with heterogeneous fiber size and shape and increased intercellular space, potentially attributed to muscle fiber atrophy during skeletal muscle aging. Under LBW intervention, the G-Aged group showed increased muscle fiber CSA (*p* = 0.2427). These results indicate that LBW intervention effectively enhances skeletal muscle mass in aged mice, significantly improves myofiber structural integrity, and demonstrates substantial potential for preventing skeletal muscle atrophy.

### 3.4. Mechanisms Underlying Effects on Aging-Related Factors

**Figure 4:**
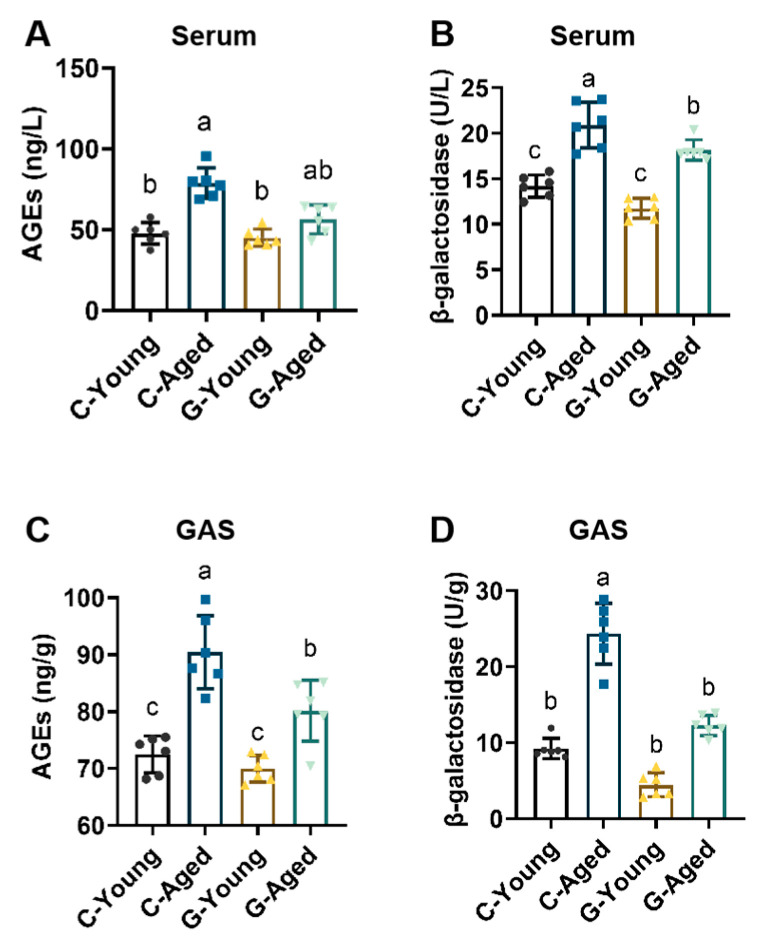
Effects of long-term LBW consumption on age-related marker expression in mice (*n* = 6): (**A**) Serum AGEs levels; (**B**) Serum β-galactosidase levels; (**C**) GAS AGEs levels; (**D**) GAS β-galactosidase levels. Significant differences were indicated by different letters (*p* < 0.05).

Expression levels of AGEs and SA-β-gal in mice of each group were detected, and the results were shown in Figure 4. Compared with C-Young, the C-Aged group exhibited significantly higher AGEs levels in serum and gastrocnemius muscle (*p* < 0.005), while the G-Young group showed no significant change (*p* = 0.9278, *p* = 0.7881, respectively). In contrast, the G-Aged group had significantly lower serum AGEs levels than the C-Aged group (*p* = 0.0004), with no significant differences in AGEs levels between the G-Aged group and C-Young group in both serum and gastrocnemius muscle (for serum, *p* = 0.2640; for GAS, *p* = 0.0437). Additionally, natural aging led to a significant increase in SA-β-gal levels in serum and gastrocnemius muscle (*p* < 0.05), whereas long-term LBW consumption effectively reduced SA-β-gal levels in these samples. These findings indicate that long-term LBW intake significantly decreases the expression of senescence markers in skeletal muscle of aged mice. Concomitant with the decline in senescence marker expression, aged mice exhibited positive improvements in functional indices such as muscle strength and endurance, findings that support a potential mechanism through which LBW attenuates skeletal muscle aging.

**Figure 5:**
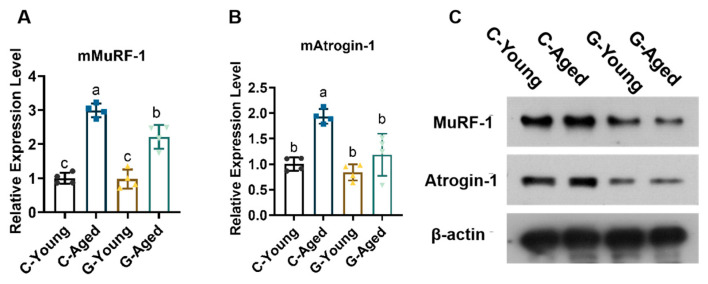
Effects of LBW consumption on FOXO signaling pathway expression in mice (*n* = 4): (**A**) mMuRF-1 relative expression; (**B**) mAtrogin-1 relative expression; (**C**) Western blot analysis of MuRF-1 and Atrogin-1 protein expression. Significant differences were indicated by different letters (*p* < 0.05).

As shown in Figure 5, real-time quantitative PCR (qRT-PCR) results demonstrated that in C-Aged mice, the mRNA expression levels of FOXO signaling pathway downstream key atrophic factors Atrogin-1 and MuRF-1 were significantly higher than those in the control group (for Atrogin-1, *p* = 0.0007; for MuRF-1, *p* < 0.0001). As core molecules mediating muscle protein degradation, the abnormally high expression of Atrogin-1 and MuRF-1 indicated that natural aging induced excessive activation of the FOXO signaling pathway, thereby leading to skeletal muscle protein metabolic disorders. In contrast, G-Aged mice exhibited significantly lower MuRF-1 expression in skeletal muscle compared to C-Aged mice (*p* = 0.0052), and Atrogin-1 expression levels more closely resembled those in the young group (*p* = 0.7798). As can be seen from the Western Blot images, the expression levels of MuRf-1 and Atrogin-1 in G-Aged and G-Young were similar, which further proved the anti-skeletal muscle aging effect of LBW. These results confirm that LBW intervention effectively inhibits excessive activation of the FOXO signaling pathway, blocks abnormal expression of atrophic factors, regulates age-related muscle protein metabolic imbalance at the molecular pathway level, and thereby alleviates skeletal muscle atrophy.

### 3.5. Mechanisms Underlying Effects on Oxidative Stress and Inflammation Levels

**Figure 6:**
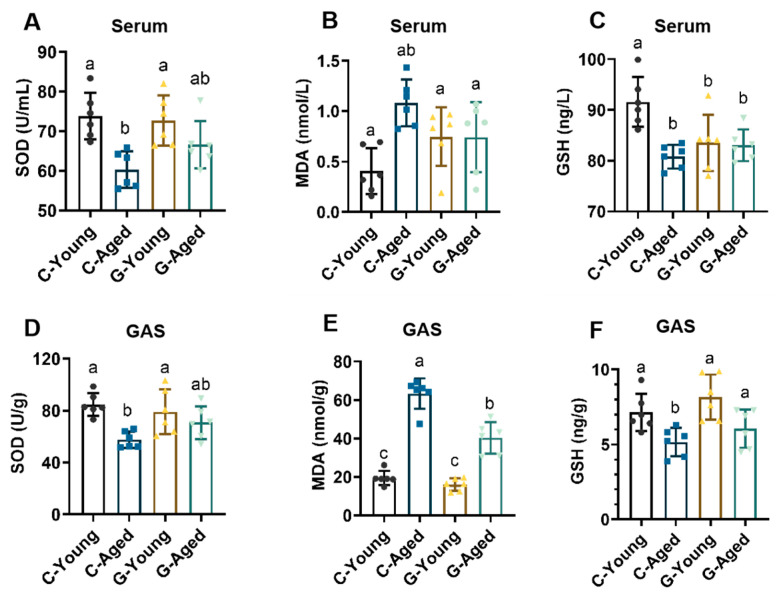
Effects of long-term LBW consumption on oxidative stress in mice (*n* = 6): (**A**) Serum MDA levels; (**B**) Serum SOD levels; (**C**) Serum GSH levels; (**D**) GAS MDA levels; (**E**) GAS SOD levels; (**F**) GAS GSH levels. Significant differences were indicated by different letters (*p* < 0.05).

Oxidative stress levels in each group were analyzed by measuring the contents of superoxide dismutase (SOD), malondialdehyde (MDA), and glutathione (GSH) in serum and GAS muscle. As shown in Figure 6, compared with the C-Young group, the C-Aged group displayed significantly lower levels of SOD and GSH (serum: SOD, *p* = 0.003; GSH, *p* = 0.0013; GAS: SOD, *p* = 0.0043) and a significantly higher MDA level in the gastrocnemius muscle (*p* < 0.0001). In contrast, the G-Young group only differed significantly from the C-Young group in terms of serum GSH levels (*p* = 0.0161). These results indicate that long-term LBW consumption has no obvious impact on oxidative stress in young mice. In contrast, the G-Aged group had significantly lower MDA levels in serum and GAS muscle than the C-Aged group (*p* < 0.05), with no significant differences in SOD and GSH levels compared to the C-Young group (for serum, *p* values for SOD were 0.1605; for GAS, *p* values for SOD and GSH were 0.2126 and 0.4610, respectively). These results suggest that long-term LBW intake exerts positive effects on skeletal muscle function by regulating the body’s antioxidant defense system, reducing oxidative damage, and serving as a key mechanism for improving muscle performance.

**Figure 7:**
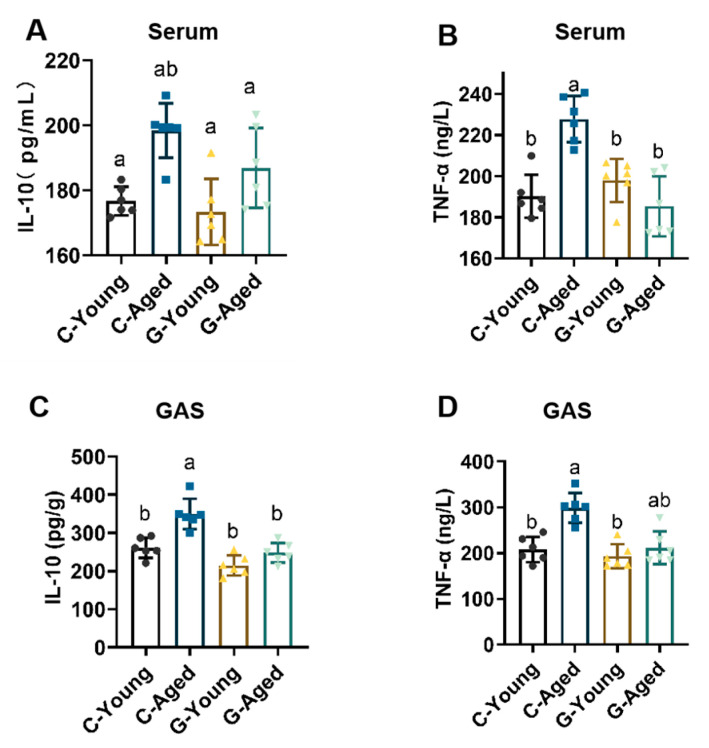
Effects of long-term LBW consumption on inflammation levels in mice (*n* = 6): (**A**) Serum IL-10 levels; (**B**) Serum TNF-α levels; (**C**) GAS IL-10 levels; (**D**) GAS TNF-α levels. Significant differences were indicated by different letters (*p* < 0.05).

Inflammation, one of the most critical biological hallmarks characterizing the aging process, was analyzed in serum and skeletal muscle of each group, as shown in Figure 7. LBW intervention significantly lowered serum levels of the pro-inflammatory cytokine TNF-α in aged mice (*p* < 0.0001), indicating that long-term LBW consumption effectively suppresses aging-associated chronic inflammation and mitigates persistent inflammatory damage to cellular and tissue structures at its source. Additionally, LBW intervention moderately reduced levels of the anti-inflammatory cytokine IL-10, preventing its excessive elevation—a state that could compromise essential immune responses (*p* < 0.0001) [39]. By tempering the concentrations of inflammatory cytokines, LBW further enhances the microenvironment of multiple systems in aged mice, thereby retarding systemic aging.

### 3.6. Metabolomics Analysis

**Figure 8:**
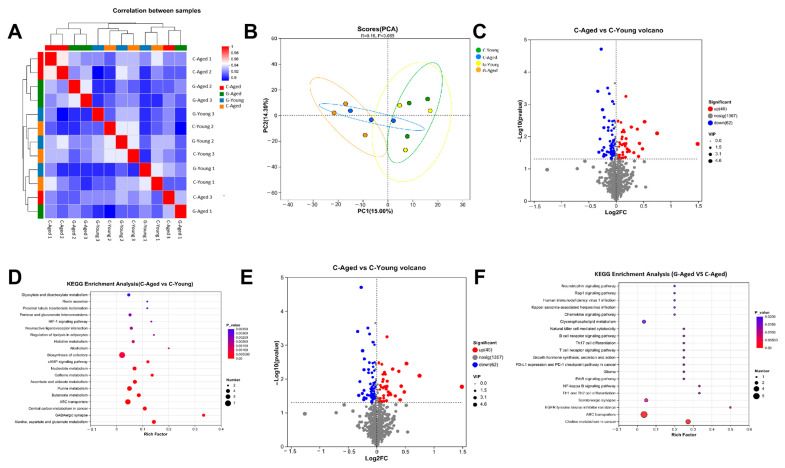
Effect of long-term LBW consumption on: (**A**) Correlation Heatmap; (**B**) Principal Component Analysis (PCA); (**C**) OPLS-DA score (C-Aged vs. C-Young); (**D**) Pathway Enrichment Analysis (C-Aged vs. C-YOUNG); (**E**) PLS-DA SCORE (G-AGED vs. C-Aged); (**F**) Pathway Enrichment Analysis (G-Aged vs. C-Aged) (*n* = 4).

Metabolomics results showed high overall correlation between samples, with all correlation coefficients exceeding 0.9 (Figure 8A). Notably, parallel samples within the same group exhibited extremely high correlation, demonstrating minimal overall sample variability and robust experimental control, which validated the reliability of the results. In contrast, the correlation coefficients between the C-Aged and G-Aged groups were below 0.93, indicating that long-term LBW consumption induced noticeable changes in metabolite profiles, leading to distinct compositional differences in metabolites between the two groups.

In the PCA score plot, samples were visualized as coordinate points after dimensionality reduction, where the Euclidean distance between points reflected metabolic differences (Figure 8B). The sample points of the C-Aged were highly clustered with minimal dispersion, indicating a high consistency in metabolic changes induced by aging. This made the C-Aged group suitable as a control to establish metabolic baselines and evaluate the effects of long-term LBW intervention. In contrast, the G-Young and C-Young groups showed nearly overlapping distributions, suggesting that long-term LBW consumption had no significant impact on metabolism in young mice.

The volcano plot of C-Aged and C-Young revealed profound metabolic perturbations associated with natural aging (Figure 8C). Specifically, a substantial number of metabolites were dysregulated: 46 metabolites were upregulated and 62 were downregulated (replace with actual counts from plot), which reflected the inherent metabolic decline during aging. In the further KEGG enrichment analysis of C-Aged vs. C-Young, HIF-1 signal pathway showed a significant difference (high Rich Factor, low *p*-value). HIF-1 signal pathway promotes the production of oxidative stress and the production of inflammatory factors through a variety of mechanisms, which further illustrates the production of oxidative stress response and inflammation by aging from a metabolic perspective [40,41].

In contrast, Figure 8E demonstrated that long-term LBW consumption remodels the aged metabolic landscape. A total of 68 metabolites were upregulated and 45 downregulated in G-Aged mice, with distinct trends counteracting age-related dysregulation. By comparing Figure 8C,E, it was obvious that LBW intervention partially reverses natural aging-induced metabolic chaos—targeting key pathways like oxidative stress, energy metabolism, and inflammatory metabolite production—to mitigate age-related decline in skeletal muscle and systemic health. Notably, KEGG enrichment analysis of the G-Aged versus C-Aged groups revealed a significant difference in the NF-κB signaling pathway (Figure 8F). The C-Aged group exhibited more pronounced activation of this pathway, as evidenced by a higher degree of enrichment. This indicated that the G-Aged group exerted an inhibitory effect on this classic pro-inflammatory pathway, which could alleviate chronic muscle inflammation in aged mice, with a statistically significant difference (*p* < 0.05) [42]. This is most likely due to the fact that the optimized LBW is rich in lycium polysaccharides, which inhibit the activation of the NF-κB pathway and reduce the transcription of pro-inflammatory factors [43]. Similarly, for the chemokine signaling pathway, the C-Aged group showed more prominent enrichment, the difference was statistically significant (*p* < 0.05). This pathway difference suggested that LBW could reduce the recruitment and infiltration of inflammatory cells into muscle tissues, improving the inflammatory microenvironment [44].

## 4. Discussion

In this study, we first optimized the processing parameters of LBW to ensure its antioxidant activity, then used 8-week-old young mice and 18-month-old aged mice as models to investigate the health effects of long-term LBW consumption. Muscle atrophy is a key determinant of systemic aging during the aging process, and maintaining skeletal muscle mass stability is critical for motor function and overall health [45,46,47]. Our experimental data showed that although long-term LBW intake had no significant effect on body weight in aged mice compared with the control group, it significantly increased muscle mass ratio, enhanced structural integrity, and improved motor capacity (as reflected by increased grip strength, rotarod fall latency, and treadmill running distance/time). These results confirm that LBW has the potential to maintain muscle mass and delay the decline of muscle function, thereby indirectly reducing the incidence of sarcopenia.

Notably, skeletal muscle function and mass are primarily determined by myofibers—the core units responsible for muscle contraction, whose status directly correlates with muscle function [48]. The significant increase in gastrocnemius myofiber cross-sectional area observed in this study suggests that myofibers are the cell type most responsive to LBW intervention. Given the improvements in myofiber morphology and downstream function induced by LBW, we hypothesize that myofibers are key target cells through which LBW counteracts age-related muscle decline.

Elevated levels of AGEs and SA-β-gal serve as important biomarkers of cellular senescence, while the expression of Atrogin-1 and MuRF-1 regulates muscle function via the FOXO signaling pathway. During aging, FOXO (particularly FOXO3) regulates Atrogin-1 and MuRF-1 to maintain muscle protein homeostasis under physiological conditions, but its excessive activation disrupts this balance and modulates energy metabolism and anti-apoptotic pathways in a pathological way, accelerating muscle aging [49]. Oxidative stress and chronic inflammation are two prominent hallmarks of aging [50,51,52,53]. This study found that long-term LBW consumption reduced AGEs and SA-β-gal levels in aged mice, and more importantly, downregulated Atrogin-1 and MuRF-1 expression by targeting the overactivated FOXO pathway in aged skeletal muscle—thereby regulating the FOXO pathway, mitigated skeletal muscle cell senescence by correcting age-related imbalances in muscle protein metabolism at the molecular level. Our results demonstrate that LBW specifically inhibits the pathological overactivation of FOXO in aged muscle, rather than altering its basal activity in young tissue.

Additionally, LBW effectively decreased oxidative stress and regulated the levels of inflammation, alleviating cellular and tissue damage and maintaining systemic homeostasis. Regulation of aging-related factors, oxidative stress, and inflammation represents the core mechanisms by which LBW improves skeletal muscle function. Metabolomics analysis revealed that LBW intervention significantly affected fatty acid biosynthesis and amino acid metabolism pathways in aged mice, suggesting a potential role in delaying skeletal muscle aging via energy metabolism regulation.

Metabolomic analysis further revealed high overall correlation among samples (all over 0.9), confirming robust experimental reliability. Long-term LBW consumption significantly remodeled the metabolic profile of aged mice, with 68 metabolites upregulated and 45 downregulated versus naturally aged controls. This intervention suppressed activation of pro-inflammatory pathways like NF-κB and chemokine signaling pathway, suggesting LBW alleviates aging-related metabolic abnormalities via multi-pathway regulation.

In summary, long-term intake of LBW exerts anti-skeletal muscle aging effects by regulating specific signaling pathways and related molecules: it downregulates the expression of downstream muscle atrophy factors (Atrogin-1 and MuRF-1) via modulation of the FOXO signaling pathway; regulates inflammation through the NF-κB pathway and chemokine signaling pathways, thereby reducing the production of pro-inflammatory cytokines; and simultaneously decreases aging-related biomarkers (AGEs, SA-β-gal), improves skeletal muscle morphology, regulates muscle weight, and enhances motor capacity. These mechanisms collectively mediate the anti-skeletal muscle aging effects of LBW. The preclinical findings presented in this study lay a foundation for its subsequent translation into practical applications in the field of food nutrition.

Compared with similar studies, this experiment uses a natural drinking water intervention method instead of traditional extract injection or gavage, which is more in line with the daily dietary intake pattern. Meanwhile, it breaks through the research limitation of single components (such as polysaccharides or polyphenols), and through the synergistic effect of multiple components in natural LBW, it is closer to the nutritional intervention effect in actual dietary scenarios. Notably, existing studies have mainly focused on single bioactive components and elaborated their mechanisms systematically—for instance, the activation of the Nrf2 antioxidant pathway by LBP has been validated in multiple experimental models, providing a clear mechanistic basis for its biological effects [54]. However, although the present study has demonstrated that LBW exerts comprehensive anti-skeletal muscle aging effects, several questions remain unanswered. Specifically, this study has not yet clarified the synergistic interaction patterns of bioactive components in LBW nor their specific regulatory sites within pathways. This provides insights for future research.

Overall, the findings of this study are expected to provide a preclinical theoretical basis for scientific and effective nutritional intervention strategies. From the perspective of translational medicine, based on its “food-medicine homology” property, we confirmed the safety of LBW through a 12-week mouse study. However, given species differences, in terms of clinical translation, deriving human-equivalent doses via body surface area conversion and conducting small-scale randomized controlled trials (RCTs) in elderly individuals at risk of sarcopenia can serve as initial feasible steps for bridging preclinical and clinical evidence.From future industrial perspective, while basic products like *Lycium barbarum* puree have been developed, commercializing LBW as a natural and efficient anti-aging beverage could fill a market gap, bridging laboratory research and daily nutrition to realize the societal value of food science innovations.

## 5. Conclusions

In conclusion, long-term consumption of LBW alleviates age-related skeletal muscle dysfunction in aged mice by modulating oxidative stress, inflammation, and muscle atrophy, likely via regulating aging-related factors (AGEs, SA-β-gal), FOXO-mediated atrophy factors (Atrogin-1, MuRF-1), and pro-inflammatory pathways (NF-κB, chemokine signaling). This natural drinking intervention, leveraging multi-component synergism, provides a practical nutritional strategy for combating skeletal muscle aging and informs the development of *Lycium barbarum*-based functional products.

## Figures and Tables

**Table 1:** Animal experimental design to explore the healthy effect of long-term drinking LBW.

Group	Age	Intervention Methods	Numbers of Animals
C-Young	8 Weeks	Drinking Water	8
C-Aged	≥18 Months	Drinking Water	8
G-Young	8 Weeks	LBW	8
G-Aged	≥18 Months	LBW	8

**Table 2:** Primer sequences.

Gene	Forward Primer (5′-3′)	Reverse Primer (5′-3′)
18S	ACCGCAGCTAGGAATAATGGA	CAAATGCTTTCGCTCTGGTC
mAtrogin-1	CGCCACTCCGGGACATAG	GAAGTCGTCTGCTGTCTCAAAGG
mMuRF-1	GAGACATCCCCCTATTTCTACCA	GCTCAGTCCGCTCATAGCC

**Table 3:** Antioxidant activity of LBW under different brewing material ratios (*n* = 4).

Free Radical	1:2	1:5	1:10	1:20
DPPH clearance activity (%)	42.67 ± 5.59 de	57.57 ± 3.43 c	74.94 ± 3.29 a	78.25 ± 6.82 a
ABTS free radical scavenging activity (%)	37.78 ± 2.40 e	42.73 ± 3.29 e	46.27 ± 4.99 d	48.27 ± 5.01 d
Hydroxyl radical scavenging clearance (%)	35.40 ± 4.01 e	54.82 ± 6.77 cd	63.22 ± 4.21 bc	71.75 ± 6.82 ab

Significant differences were indicated by different letters (*p* < 0.05).

**Table 4:** Antioxidant activity of LBW at different brewing temperatures (*n* = 4).

Free Radical	40 °C	60 °C	80 °C	100 °C
DPPH clearance activity (%)	63.60 ± 4.26 b	66.90 ± 6.11 ab	72.79 ± 3.16 a	50.76 ± 6.35 c
ABTS free radical scavenging activity (%)	47.30 ± 1.32 d	50.30 ± 6.19 cd	63.38 ± 2.15 b	46.58 ± 1.91 d
Hydroxyl radical clearance (%)	39.70 ± 3.11 e	61.85 ± 4.57 b	67.40 ± 4.39 a	59.70 ± 3.27b c

Significant differences were indicated by different letters (*p* < 0.05).

**Table 5:** Fundamental Component Analysis (*n* = 4).

Component Name	LBW
Polysaccharide content (mg GLU/g)	71.81 ± 2.76
Anthocyanin content (mg/100 g)	21.13 ± 3.52
Total phenol content (mg GAE/g)	18.80 ± 1.14
Total flavonoid content (mg CAE/g)	2.37 ± 0.39
Carotenoid content (mg/100 g)	9.08 ± 1.22
Betaine content (mg/100 g)	41.31 ± 6.82

## Data Availability

The original contributions presented in this study are included in the article. Further inquiries can be directed to the corresponding authors.
